# Simplified and Rapid Preparation Protocol for Producing *Aloe Vera*-Based Natural Coagulant for Water Treatment

**DOI:** 10.3390/mps9040106

**Published:** 2026-07-08

**Authors:** Danieli Soares de Oliveira, Clainer Bravin Donadel

**Affiliations:** 1 Federal Institute of Espírito Santo - Campus Cariacica, Rodovia Governador José Sette 184, Cariacica 29150-410, Espírito Santo, Brazil; danieli@ifes.edu.br; 2 Federal Institute of Espírito Santo - Campus Vitória, Av. Vitória 1729, Vitória 29040-780, Espírito Santo, Brazil

**Keywords:** *Aloe vera*-based coagulant, natural coagulants, turbidity removal, decentralized water treatment, simplified water treatment

## Abstract

Natural coagulants have emerged as potential alternatives to synthetic chemicals in water treatment, especially for decentralized and low-resource applications. However, many previously reported *Aloe vera*-based coagulant preparation methods rely on drying, powder production, distilled water extraction, refrigeration, or other laboratory-dependent procedures that increase operational complexity and limit practical implementation. This study presents a simplified and rapid protocol for producing an *Aloe vera*-based natural coagulant using accessible materials and simplified preparation steps. The proposed methodology consists of extracting *Aloe vera* g13el, homogenizing 2 g of fresh gel with 50 mL of tap water using a household blender, and applying simple paper filtration to obtain the liquid coagulant. The protocol can be completed in less than 10 min without specialized laboratory infrastructure, energy-intensive processing, or laboratory-grade reagents. Coagulation performance was evaluated using synthetic turbid water with initial turbidity levels of 100, 200, and 300 NTU. Significant turbidity reduction was observed under all tested conditions, with several samples reaching residual turbidity values close to or equal to 0 NTU after 50–60 min of sedimentation. The results demonstrate the potential of the proposed protocol as a rapid, reproducible, and accessible approach for future investigation in point-of-use and decentralized water treatment applications.

## 1. Introduction

Access to safe drinking water remains a major global challenge, particularly in decentralized and low-resource settings where conventional water treatment systems are often economically or technically unfeasible. Rapid urbanization, industrialization, population growth, and inadequate wastewater disposal have intensified the degradation of water resources, especially surface waters used for public supply, increasing turbidity, color, organic matter, pathogens, and suspended solids in raw water sources [1,2].

Among the physicochemical processes employed for water treatment, coagulation–flocculation–sedimentation is one of the most widely used due to its operational simplicity, relatively low cost, and high efficiency in destabilizing colloidal particles and reducing water turbidity. Conventional coagulation processes are commonly performed using metal-based coagulants such as aluminum sulfate and ferric chloride. Although effective, these coagulants present important limitations, including the generation of large sludge volumes, alteration of water pH and alkalinity, high operational costs, and concerns regarding residual metal concentrations in treated water. In addition, excessive aluminum exposure has been associated with neurotoxic effects and possible links to neurological disorders [3,4,5].

In response to these limitations, increasing attention has been directed toward the development of sustainable, biodegradable and environmentally friendly natural coagulants derived from plant-based materials. Recent review studies highlighted the growing interest in natural coagulants for water and wastewater treatment applications due to their renewability, reduced toxicity, lower sludge production, and potential applicability in decentralized and low-resource settings [1,6,7]. Plant-based coagulants such as *Moringa oleifera*, cactus, acorn, banana peel, citrus peel, tamarind seeds, sorghum, and *Aloe vera* have demonstrated promising results for turbidity reduction and pollutant removal in different water treatment scenarios [3,4,6,8,9,10].

Among these alternatives, *Aloe vera* has emerged as a promising natural coagulant due to its worldwide availability, low cost, biodegradability, and presence of active functional groups capable of destabilizing colloidal particles. Previous studies demonstrated the successful application of *Aloe vera* in drinking water treatment, surface water clarification, and wastewater treatment processes [3,4,11]. Reported applications include turbidity reduction, color removal, organic matter reduction, coagulation aid applications, and hybrid coagulation systems involving *Aloe vera* combined with other natural coagulants such as *Moringa oleifera* and sorghum [11].

The coagulation performance of *Aloe vera* is associated with the presence of proteins, polysaccharides, carbohydrates, amides, hydroxyl, and carboxylic functional groups, which act through mechanisms such as adsorption, charge neutralization, intermolecular hydrogen bonding, and polymer bridging [3,4].

Several studies have demonstrated significant turbidity reduction efficiencies using *Aloe vera*-based coagulants under different operational conditions. Authors in [3] reported turbidity reductions close to 88% using *Aloe vera* liquid extracts in drinking water treatment. Other studies have investigated the combined use of *Aloe vera* and *Moringa oleifera* for the removal of turbidity, color, COD, phosphate, and dissolved solids from surface waters [11].

Despite the promising results reported in the literature, the preparation methodologies adopted for *Aloe vera*-based coagulants vary considerably among studies. Different protocols involve drying, grinding, sieving, solvent extraction, chemical activation using NaCl, NaOH, or HCl, filtration steps, centrifugation, magnetic stirring, and extraction using distilled water or chemical solvents [3,4]. In many cases, powdered materials are produced through drying and heating stages prior to extraction, increasing operational complexity and energy demand. In contrast, fresh gel extraction approaches may reduce processing requirements and simplify local preparation procedures. Moreover, fresh-use preparation strategies may help preserve the activity of coagulating compounds while simplifying local production. Some studies have also employed response surface methodology (RSM), optimization procedures, and multiple operational variables to improve coagulation efficiency [11]. Furthermore, recent review papers highlighted that the performance of *Aloe vera*-based materials is strongly influenced by the adopted preparation methodology, while the lack of standardized extraction protocols remains one of the major barriers for reproducibility, scalability, and large-scale implementation of natural coagulants [1,6]. In addition, most studies have focused on optimizing coagulation performance rather than simplifying coagulant preparation procedures, resulting in protocols that often remain dependent on laboratory infrastructure and specialized processing steps.

In many cases, previously reported preparation methods require laboratory infrastructure, controlled drying conditions, chemical reagents, multiple extraction stages, or specialized equipment, which may limit their practical application in vulnerable, decentralized, rural, or low-resource settings [4,7]. Preparation times reported in the literature may range from several hours to multiple days due to drying, powder production, extraction, or storage stages, creating additional barriers for rapid local implementation. This limitation becomes particularly relevant when considering household-level or community-scale water treatment applications, especially in regions with restricted technical infrastructure.

Therefore, there remains a need for simplified, rapid, and reproducible preparation methodologies capable of producing effective *Aloe vera*-based coagulants using minimal infrastructure and operational complexity. The novelty of the present work resides not in the individual preparation steps themselves, but in the development and experimental validation of an operationally simplified preparation protocol capable of producing an effective *Aloe vera*-based coagulant while substantially reducing preparation complexity compared with previously reported methodologies. The proposed protocol eliminates drying, heating, chemical extraction, distilled water requirements, and other laboratory-dependent procedures commonly reported in the literature while maintaining turbidity removal efficiencies comparable to those reported for previously published *Aloe vera* preparation methodologies. In this context, the present study proposes and experimentally evaluates a simplified preparation protocol for producing an *Aloe vera*-based natural coagulant using synthetic turbid water as a model system for preliminary coagulation assessment. The proposed approach prioritizes operational simplicity, reduced processing steps, and minimal energy requirements by eliminating drying, heating, powder production, distilled water extraction, storage, and other laboratory-dependent stages commonly reported in previous studies. The protocol was designed to be completed in less than 10 min using accessible materials and straightforward operational steps. The proposed protocol also enables immediate coagulant application after preparation, without storage or stabilization steps, while maintaining turbidity removal efficiencies comparable to those reported for substantially more complex preparation methods.

## 2. Protocol for Preparing the *Aloe Vera*-Based Natural Coagulant

**Figure 1:**
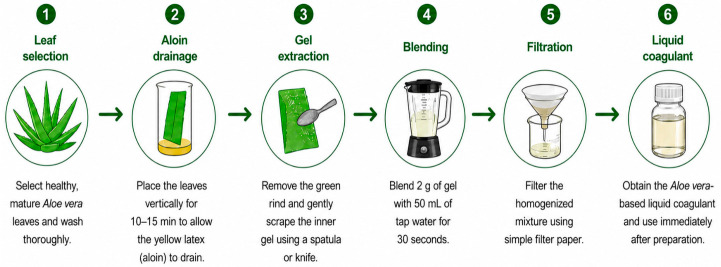
Schematic workflow of the *Aloe vera*-based coagulant preparation protocol.

This section describes the protocol developed for producing an *Aloe vera*-based natural coagulant using accessible materials and simplified methodological steps. The methodology was designed to minimize infrastructure requirements while maintaining rapid preparation and operational reproducibility. The protocol consists of *Aloe vera* gel extraction, homogenization with tap water, simple filtration, and immediate application of the liquid coagulant in coagulation tests. A schematic overview of the proposed preparation protocol is presented in Figure 1. The materials and equipment required for the procedure are presented in Table 1 and Table 2, respectively, while the complete step-by-step preparation protocol is described in Table 3.

## 3. Results and Discussion

This section presents the experimental evaluation of the *Aloe vera*-based natural coagulant under different initial turbidity conditions. The coagulation performance was assessed through jar test experiments using synthetic turbid water prepared with bentonite suspensions. Turbidity reduction behavior, optimal dosage ranges, and sedimentation performance are discussed for initial turbidity levels of 100, 200, and 300 NTU.

### 3.1. Preparation of Synthetic Turbid Water and Jar Test Procedure

To validate the alternative method for producing the *Aloe vera*-based natural coagulant, turbidity removal efficiency was used as the primary performance indicator. Turbidity is a key parameter in assessing the effectiveness of coagulation, flocculation, and sedimentation processes in water treatment systems.

All coagulation trials were carried out using a jar test apparatus, which allowed the simultaneous evaluation of different operational conditions, including coagulant concentration, under controlled laboratory settings. The experiments were conducted using a jar test apparatus (JTAT6J2LDIG-CS, Athon, Barueri, Brazil), while turbidity measurements were performed using a calibrated nephelometric turbidimeter (TU430, AKSO, São Leopoldo, Brazil). Additionally, pH monitoring was carried out using a Milwaukee Instruments pH meter (MW150 MAX, Milwaukee Instruments, Rocky Mount, NC, USA).

The test procedure began with the preparation of turbid water samples obtained by adding bentonite to tap water. The suspension was prepared directly in 2 L jars by weighing and dispersing bentonite to reach target turbidity levels of 100, 200, and 300 NTU. The mixture was then stirred at 500 rpm for 30 min to ensure homogeneous dispersion of the bentonite particles.

After homogenization, the synthetic turbid water was transferred to storage containers and allowed to rest for at least 24 h, following recommendations from previous studies [12]. This stabilization period aimed to ensure complete hydration of the clay particles and improve the reproducibility of the experimental conditions before coagulation tests.

The prepared synthetic water was subsequently used in coagulation experiments with the *Aloe vera*-based coagulant, following standard jar test procedures to evaluate its performance under the defined operational conditions.

Different dosages of the *Aloe vera*-based natural coagulant were added to each jar containing the prepared turbid water. The samples were then subjected to rapid mixing to ensure homogeneous coagulant dispersion and initiate floc formation. This initial stage was essential to promote effective interaction between suspended particles and the coagulant during the destabilization process.

To prepare the coagulant solution, 2 g of *Aloe vera* gel was diluted in 50 mL of tap water and homogenized using a household blender. The operational parameters adopted for the coagulation/flocculation stage consisted of rapid mixing at 151 rpm for 1.11 min, according to the procedure described in [11].

After rapid mixing, the samples were immediately subjected to sedimentation, allowing the flocs to settle and promoting impurity removal from the water. Residual turbidity was measured at predefined sedimentation intervals, providing a quantitative assessment of the coagulant performance under the tested conditions.

All turbidity measurements and experimental conditions were evaluated in triplicate to ensure reproducibility and reliability of the results. Experimental results are reported as mean values obtained from triplicate measurements. The corresponding standard deviations were calculated to quantify experimental variability and assess the reproducibility of the proposed coagulant preparation protocol. Standard deviation values for all experimental conditions are presented in Appendix A. Throughout the experiments, the pH remained stable between 6.9 and 7.2, indicating that the *Aloe vera*-based natural coagulant did not significantly alter the physicochemical characteristics of the treated water.

It is important to highlight that the physicochemical properties of *Aloe vera* gel, including polysaccharide concentration, viscosity, and moisture content, may vary depending on environmental conditions, cultivation practices, and plant maturity. These variations can influence coagulant behavior and treatment performance, representing an important factor for future protocol standardization and large-scale applications.

### 3.2. Turbidity Removal in Samples with an Initial Turbidity of 300 NTU

The turbidity removal performance obtained for water samples with an initial turbidity of approximately 300 NTU is presented in Table 4. Overall, the *Aloe vera*-based natural coagulant promoted rapid clarification, with substantial turbidity reductions occurring during the first stages of sedimentation.

After 10 min of settling, residual turbidity values ranged from 32.37 NTU (Jar #2) to 16.67 NTU (Jar #6), corresponding to turbidity reductions greater than 89% for all evaluated dosages. These results demonstrate the rapid action of the coagulant during the initial destabilization and floc formation stages.

As sedimentation progressed, turbidity values continued to decrease significantly. After 20 min, residual turbidity ranged from 14.96 NTU (Jar #1) to 2.02 NTU (Jar #6), while after 30 min, Jar #6 had already reached complete clarification (0.00 NTU). The remaining jars also exhibited progressive clarification, with residual turbidity values below 12 NTU at this stage.

From 40 min onward, the clarification process became more stable, with only minor variations observed between subsequent measurements. Jars #5 and #6 achieved complete clarification from 50 min onward and maintained residual turbidity equal to 0.00 NTU until the end of the experiment (60 min). The remaining jars also showed low residual turbidity values at the end of the settling period, ranging from 6.61 NTU (Jar #1) to 1.44 NTU (Jar #4).

The results indicate that higher coagulant dosages promoted faster clarification and lower final turbidity values under high initial turbidity conditions. Among the evaluated operational conditions, the best performance was observed for dosages between 115 mL and 120 mL (Jars #5 and #6), which consistently produced the lowest residual turbidity values throughout the experiment and achieved complete clarification more rapidly. These results suggest that this dosage range represents the most effective operational condition for treating water samples with initial turbidity close to 300 NTU using the proposed *Aloe vera*-based coagulant preparation protocol.

### 3.3. Turbidity Removal in Samples with an Initial Turbidity of 200 NTU

The turbidity removal results obtained for water samples with an initial turbidity of approximately 200 NTU are presented in Table 5. Similarly to the behavior observed for samples with higher initial turbidity, the *Aloe vera*-based natural coagulant promoted rapid clarification during the first stages of sedimentation.

After 10 min of settling, residual turbidity values ranged from 29.09 NTU (Jar #1) to 8.19 NTU (Jar #6), corresponding to turbidity reductions greater than 85% for all evaluated dosages. These results confirm the rapid coagulation and flocculation action promoted by the *Aloe vera*-based coagulant under intermediate turbidity conditions.

A progressive reduction in turbidity was observed throughout the sedimentation period. After 20 min, residual turbidity values decreased to between 16.13 NTU (Jar #1) and 0.00 NTU (Jar #6), with Jar #6 already reaching complete clarification at this stage. At 30 min, turbidity values ranged from 12.27 NTU (Jar #1) to 0.00 NTU (Jar #6), while at 40 min, Jars #5 and #6 both exhibited complete clarification.

From 50 min onward, all evaluated jars reached residual turbidity equal to 0.00 NTU and maintained complete clarification until the end of the experiment (60 min). These results demonstrate the high effectiveness of the proposed coagulant preparation protocol for intermediate turbidity conditions and indicate strong sedimentation stability after floc formation.

The best operational performance was observed for coagulant dosages between 100 mL and 105 mL (Jars #5 and #6), which consistently achieved complete clarification more rapidly than the remaining conditions. Compared with the experiments conducted at 300 NTU, lower coagulant dosages were sufficient to achieve complete turbidity removal, suggesting a direct relationship between optimal coagulant dosage and initial turbidity level. These results reinforce the applicability of the proposed *Aloe vera*-based coagulant for different turbidity conditions using relatively simple dosage adjustments.

### 3.4. Turbidity Removal in Samples with an Initial Turbidity of 100 NTU

The turbidity removal behavior observed for water samples with an initial turbidity of approximately 100 NTU is presented in Table 6. The results demonstrate that the *Aloe vera*-based natural coagulant remained effective even under lower initial turbidity conditions, promoting progressive clarification throughout the sedimentation period.

After 10 min of settling, residual turbidity values ranged from 25.46 NTU (Jar #1) to 14.21 NTU (Jar #6), indicating effective particle destabilization and floc formation across all evaluated dosages. Although clarification occurred slightly more gradually compared with the experiments conducted at higher initial turbidity levels, substantial turbidity reductions were still observed during the initial sedimentation stage.

At 20 min, residual turbidity values further decreased to between 10.81 NTU (Jar #1) and 4.09 NTU (Jar #6). After 30 min, several samples had already reached near-zero turbidity conditions, with values ranging from 3.44 NTU (Jar #1) to 0.00 NTU (Jar #4). This progressive reduction demonstrates the ability of the coagulant to promote effective clarification even when lower concentrations of suspended particles are present.

By 40 min, most jars exhibited turbidity values close to or equal to zero. Complete clarification was achieved in all evaluated conditions from 50 min onward, with residual turbidity remaining at 0.00 NTU until the end of the experiment (60 min). These results confirm the stability of the sedimentation process and the effectiveness of the proposed coagulant preparation protocol under low turbidity conditions.

The best operational performance was observed for coagulant dosages between 65 mL and 75 mL (Jars #4, #5, and #6), which achieved complete clarification more rapidly and maintained stable residual turbidity values throughout the final settling stages. Compared with the experiments conducted at 200 and 300 NTU, lower coagulant dosages were sufficient to achieve complete clarification, reinforcing the relationship between optimal dosage and initial turbidity level. Overall, the results demonstrate the adaptability of the proposed *Aloe vera*-based coagulant preparation protocol across different turbidity conditions through relatively simple dosage adjustments.

### 3.5. Operational and Methodological Considerations

The results obtained in this study demonstrate the strong potential of the proposed *Aloe vera*-based natural coagulant preparation protocol for turbidity reduction under different initial turbidity conditions. Across all evaluated scenarios (100, 200, and 300 NTU), rapid clarification behavior was observed, with substantial turbidity reductions occurring during the first 10 to 20 min of sedimentation. In most experimental conditions, residual turbidity values reached or approached 0.00 NTU by the end of the settling period, indicating stable clarification performance over time. The rapid clarification behavior observed in this study is consistent with previous investigations involving *Aloe vera*-based coagulants reported in the literature [3,4].

The standard deviations obtained from triplicate measurements were generally higher during the initial settling stages and progressively decreased as sedimentation advanced. This behavior is consistent with the dynamic nature of floc formation and settling processes and indicates satisfactory experimental reproducibility, particularly during the final clarification stages when residual turbidity values approached zero.

To contextualize the performance of the proposed methodology, the coagulant produced using the proposed protocol was compared with previously reported *Aloe vera*-based coagulants in terms of both turbidity removal efficiency and preparation methodology. In [13] the authors reported turbidity removals of approximately 88% using liquid *Aloe vera* extracts prepared with distilled water under optimized pH conditions, whereas in [14] the authors achieved removals approaching 100% in bentonite suspensions using *Aloe vera*-based bio-coagulants. More recently, authors in [15] reported turbidity reductions of approximately 85% during river water treatment. The results obtained in the present study, with turbidity removals ranging from approximately 95% to 100% and residual turbidity values close to or equal to 0 NTU under several experimental conditions, demonstrate that the proposed protocol provides coagulation performance comparable to the highest turbidity removal efficiencies reported in the literature.

Although the present study focused on protocol development and performance evaluation rather than detailed physicochemical characterization of the produced coagulant, the coagulation activity of *Aloe vera* has been associated in previous studies with the presence of proteins, polysaccharides, carbohydrates, and functional groups such as hydroxyl, carboxyl, and amide groups [13,16]. These constituents have been reported to contribute to particle destabilization through mechanisms including charge neutralization, adsorption, intermolecular hydrogen bonding, and polymer bridging. Therefore, the turbidity reductions observed in the present study may be associated with the preservation of these bioactive compounds during the simplified preparation procedure. Future studies should further characterize the produced coagulant and investigate the specific contribution of individual compounds to coagulation performance.

The experimental results also demonstrated a consistent relationship between optimal coagulant dosage and initial turbidity level. Higher initial turbidity conditions required larger coagulant dosages to achieve complete clarification, while lower turbidity conditions were effectively treated using smaller dosages. This behavior indicates that the proposed methodology can be adapted to different operational conditions through relatively simple dosage adjustments.

Beyond coagulation performance, the proposed methodology presents important operational advantages related to simplicity, accessibility, and reproducibility compared with several *Aloe vera* preparation methods reported in the literature.

Several *Aloe vera*-based treatment methods reported in the literature involve additional processing steps, longer preparation times, and greater infrastructure requirements. For example, authors in [17] prepared *Aloe vera* biomass through washing, cutting, oven drying at 60 °C for three days, grinding, and sieving before its use as a coagulation aid. Similarly, in [18] the authors extracted the gel using distilled water, followed by magnetic stirring for 30 min, sieving, and refrigerated storage prior to application. In [19] the authors prepared *Aloe vera* powder by drying the gel at 100–105 °C for 24 h, followed by dilution in distilled water, magnetic stirring, and filtration prior to application. These procedures required preparation times ranging from several hours to multiple days and relied on laboratory infrastructure such as ovens, magnetic stirrers, refrigeration systems, distilled water, or additional processing equipment. In contrast, the protocol proposed in the present study can be completed in less than 10 min using only fresh *Aloe vera* gel, tap water, a household blender, and paper filtration. The use of tap water instead of distilled water further enhances the applicability of the protocol under real-world conditions, particularly in rural, decentralized, or low-resource environments where laboratory-grade supplies may not be readily available.

The protocol eliminates drying, heating, chemical extraction, and specialized laboratory procedures commonly associated with conventional *Aloe vera* extraction methods reported in the literature. In addition, the use of fresh *Aloe vera* gel combined with household blending and simple paper filtration significantly reduces infrastructure requirements, preparation time, and operational complexity. Unlike protocols involving drying and powder production, the proposed methodology also reduces energy demand during coagulant preparation.

Another relevant aspect concerns the operational accessibility of the protocol. The preparation procedure involves straightforward steps, including leaf selection, gel extraction, homogenization with tap water, and simple filtration, which can be performed using accessible materials and non-specialized equipment. The complete preparation process can be concluded in less than 10 min, enabling immediate coagulant application without storage or stabilization stages. The fresh-use preparation strategy may additionally help preserve the activity of coagulating compounds by reducing degradation processes associated with prolonged storage. However, the storage stability and shelf life of the prepared coagulant were not evaluated in the present study. While immediate preparation and use may be advantageous for decentralized and household-level applications, future studies should investigate storage conditions, long-term stability, and potential stabilization strategies to support broader implementation scenarios, including centralized and larger-scale treatment systems. Such investigations would help determine whether the coagulant can be prepared in advance while maintaining its coagulation performance over time.

From a methodological perspective, the use of defined preparation parameters and simplified operational steps may contribute to improved reproducibility and facilitate future protocol standardization studies involving *Aloe vera*-based coagulants. In addition, the absence of chemical solvents and activation reagents reduces the generation of secondary chemical residues during coagulant preparation. The methodological simplicity of the protocol may therefore facilitate adaptation across different geographic, environmental, and operational contexts, particularly in decentralized or low-resource scenarios.

Despite the use of standardized preparation conditions, some variability may still arise from differences in the physicochemical composition of *Aloe vera* leaves. Previous studies have highlighted that the coagulation and flocculation performance of *Aloe vera* is closely related to its bioactive constituents, including polysaccharides, proteins, and other functional compounds, and that treatment efficiency may vary according to material preparation methods [16]. Consequently, factors such as plant age, seasonal variation, climatic conditions, soil characteristics, irrigation practices, cultivation methods, and storage conditions of the harvested leaves may influence the concentration of these active compounds, potentially altering the composition of the produced coagulant and, therefore, affecting its coagulation performance. Future studies should investigate the influence of these factors on coagulant preparation, composition, and treatment efficiency, contributing to the development of more robust standardization procedures for *Aloe vera*-based water treatment applications.

The present study focused on turbidity reduction as the primary indicator of coagulation performance. However, additional water quality parameters should be considered in future investigations to further assess the practical applicability of the proposed protocol. Previous studies (e.g., [16,18]) have reported that *Aloe vera*-based treatment systems may contribute to improvements in multiple water quality parameters beyond turbidity, including color, total suspended solids (TSS), total dissolved solids (TDS), chemical oxygen demand (COD), biochemical oxygen demand (BOD), and heavy metal concentrations, depending on the treated water matrix and operational conditions. In particular, authors in [18] reported simultaneous reductions in turbidity, color, TSS, COD, and BOD during textile wastewater treatment using *Aloe vera*-derived materials. Because the present validation was performed using synthetic turbid water under controlled laboratory conditions, further investigations involving natural surface waters, groundwater, and other real water matrices are necessary to evaluate the influence of dissolved organic matter, ions, microorganisms, and other naturally occurring constituents on coagulation performance. Therefore, investigations employing real water matrices should evaluate the influence of the proposed protocol on a broader range of physicochemical and microbiological parameters, including color, dissolved organic matter, residual organic compounds, and microbial indicators, when applicable, to provide a more comprehensive assessment of treatment performance under practical conditions.

While the proposed protocol was primarily designed for decentralized, household-level, and low-resource applications, the preparation strategy may also present potential for adaptation to larger treatment scales. The individual processing steps involved in the protocol, including gel extraction, mechanical homogenization, and filtration, are based on simple physical operations that could be implemented using larger-capacity mixing and separation equipment. However, industrial or semi-industrial applications would require additional investigations addressing raw material supply, process standardization, storage stability, continuous production systems, and economic feasibility. Therefore, future studies should evaluate the scalability of the proposed methodology under pilot-scale and industrial operating conditions.

Despite the promising results obtained, some limitations of the proposed methodology should be acknowledged. First, the protocol relies on the use of fresh *Aloe vera* gel, which may limit storage, transportation, and long-term availability compared with dried or powdered coagulant formulations. Second, variations in plant composition associated with age, cultivation practices, and environmental conditions may influence coagulant performance. In addition, the protocol was validated using synthetic turbid water under controlled laboratory conditions, and its performance in real water matrices containing complex mixtures of organic matter, microorganisms, and dissolved constituents remains to be investigated. Furthermore, the present study focused primarily on turbidity reduction, while additional water quality parameters and coagulant stability during storage were not evaluated. Addressing these limitations will be important for future protocol optimization and broader implementation.

Overall, the combination of rapid preparation, minimal infrastructure requirements, reduced energy demand, and effective turbidity removal demonstrates the potential of the proposed methodology as a promising alternative for decentralized water treatment applications. However, additional investigations involving real water matrices, coagulant stability, raw material variability, and pilot-scale validation are necessary before broader implementation can be recommended. The results support future investigations involving real water matrices, pilot-scale validation, and broader applications of *Aloe vera*-based natural coagulants in simplified water treatment systems.

## 4. Study Limitations and Future Perspectives

Although the proposed protocol demonstrated promising results for turbidity reduction under controlled laboratory conditions, some limitations should be acknowledged. The coagulation tests were performed using synthetic turbid water prepared with bentonite, which does not fully represent the physicochemical complexity of natural water matrices. Therefore, additional validation using surface water, groundwater, wastewater, or waters containing natural organic matter and microorganisms is still required.

The present study focused primarily on turbidity removal as the main performance indicator. Other important water quality parameters, including organic matter, color, microbial contamination, dissolved solids, and residual organic compounds, were not evaluated and should be investigated in future studies to provide a more comprehensive assessment of the coagulant performance. Further physicochemical characterization of the prepared coagulant may also contribute to a deeper understanding of the coagulation mechanisms involved.

In addition, the proposed protocol was designed for immediate coagulant preparation and application. Although this fresh-use approach may help preserve the activity of coagulating compounds while simplifying local production, the long-term stability and shelf life of the prepared coagulant were not assessed. Future investigations should therefore evaluate storage conditions, preservation strategies, and the influence of environmental factors on the physicochemical properties of *Aloe vera* gel. Future studies should also investigate the influence of plant maturity, cultivation conditions, and seasonal variability on coagulant performance and protocol reproducibility.

Another important aspect concerns scalability and operational adaptation. The experiments were conducted at laboratory scale using jar test procedures, and additional studies are necessary to evaluate the applicability of the protocol under pilot-scale, continuous-flow, or real-world operating conditions. The need for immediate preparation and application may also represent an operational limitation in certain large-scale scenarios. Future investigations may additionally explore process optimization, dosage standardization, and integration with other decentralized water treatment technologies.

Despite these limitations, the present study provides an important methodological contribution by proposing a simplified, rapid, and reproducible protocol for preparing an *Aloe vera*-based natural coagulant using minimal infrastructure and accessible materials. The results obtained support further investigation and future validation of the proposed methodology in broader water treatment scenarios.

## 5. Conclusions

This study presented a rapid, simplified, and reproducible protocol for producing an *Aloe vera*-based natural coagulant for water treatment applications. The proposed methodology was specifically designed to minimize infrastructure requirements and operational complexity by eliminating drying, heating, chemical reagents, chemical extraction, and specialized laboratory procedures commonly reported in previous studies. Using only fresh *Aloe vera* gel, tap water, household blending, and simple paper filtration, the protocol can be completed in less than 10 min and enables immediate coagulant application after preparation, without storage or stabilization stages.

The experimental results demonstrated that the coagulant obtained through the proposed protocol was effective in reducing turbidity in synthetic water samples with initial turbidity levels of 100, 200, and 300 NTU. Rapid clarification was observed in all evaluated conditions, with substantial turbidity reductions occurring during the first stages of sedimentation and several samples reaching residual turbidity values close to or equal to 0 NTU after 50–60 min. These results demonstrate the technical feasibility of the proposed preparation method and confirm the coagulation potential of fresh *Aloe vera* gel under the tested conditions. The fresh-use preparation strategy may also help preserve the activity of coagulating compounds by avoiding prolonged storage prior to application.

Beyond its coagulation performance, the proposed protocol presents important practical and methodological advantages related to accessibility, simplicity, and reproducibility. The use of readily available materials, defined preparation parameters (2 g of gel, 50 mL dilution, and 30 s homogenization), and simplified operational steps may facilitate methodological standardization and improve reproducibility in future studies involving *Aloe vera*-based coagulants. In addition, the protocol was designed to be easily reproduced using minimal infrastructure and non-specialized equipment, increasing its potential for methodological transferability across different operational contexts.

The main contribution of this study lies in the development and preliminary validation of a simplified preparation protocol capable of producing an effective natural coagulant while significantly reducing processing stages, energy demand, and infrastructure requirements compared with conventional *Aloe vera* extraction methods described in the literature. The simplicity of the protocol may also facilitate future adaptation for pilot-scale, household-level, or community-scale water treatment applications.

Nevertheless, some limitations should be acknowledged. The experiments were performed using synthetic turbid water under controlled laboratory conditions, and additional studies are still necessary to evaluate the coagulant performance in natural water matrices containing organic matter, microorganisms, and other contaminants. Future investigations should also assess storage stability, shelf life, large-scale applicability, and the influence of environmental factors on the physicochemical properties of the *Aloe vera* gel.

Overall, the proposed protocol represents a practical contribution toward the development of standardized, accessible, and rapidly deployable natural coagulant preparation methodologies for decentralized water treatment applications.

## Figures and Tables

**Table 1:** Materials used for preparing the *Aloe vera*-based natural coagulant.

Material	Description	Illustrative Image
*Aloe vera* leaf	Plant-based raw material used as the source of gel with coagulating properties, obtained from a plant cultivated on the premises of Campus Cariacica, Espírito Santo, Brazil.	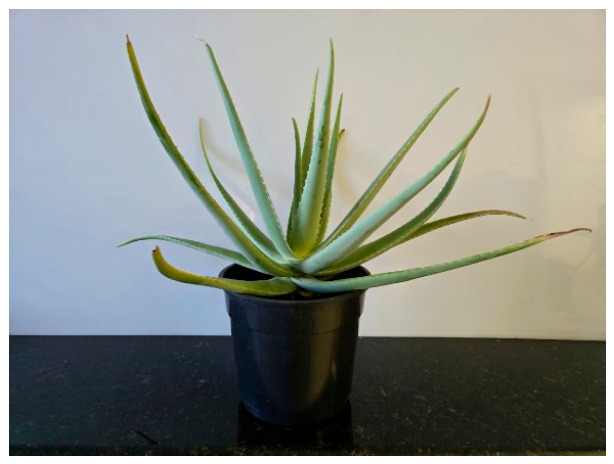
Tap water	Used to dilute the *Aloe vera* gel during the blending step.	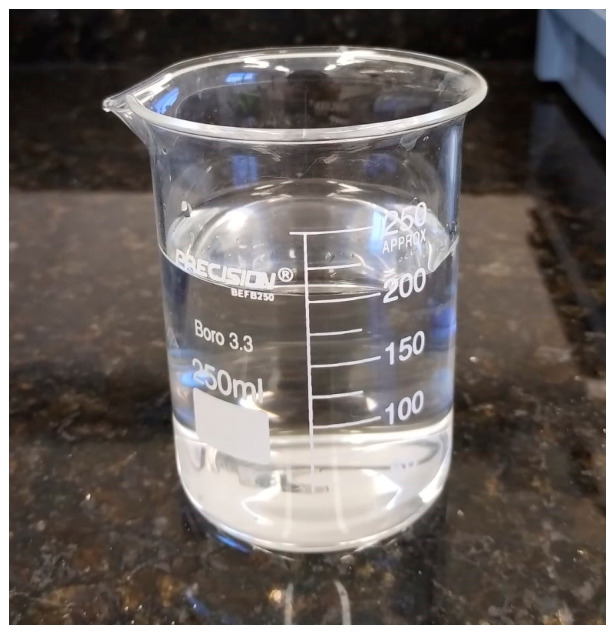
Filter paper	Used to perform the simple filtration step after blending the gel through a No. 102 paper coffee filter (Melitta, São Paulo, Brazil).	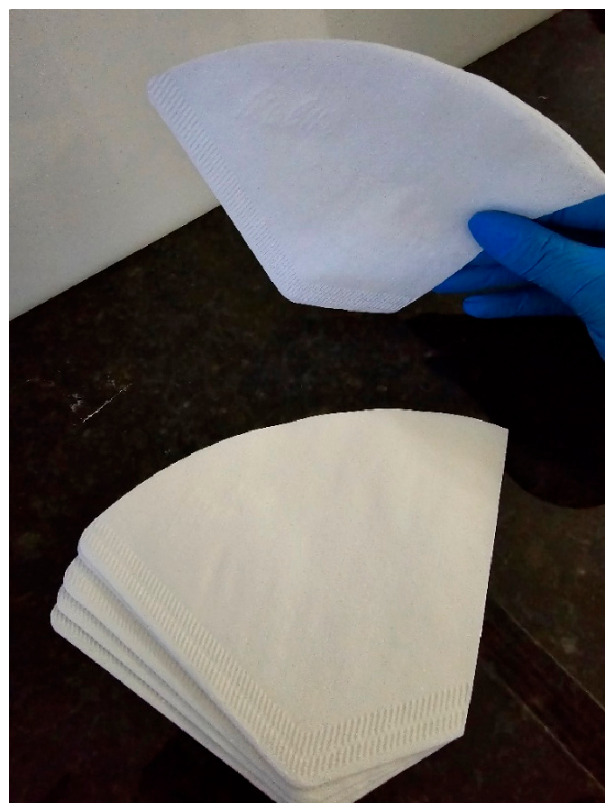

**Table 2:** Equipment used for preparing the *Aloe vera*-based natural coagulant.

Equipment	Description	Illustrative Image
Blender	Used to homogenize the *Aloe vera* gel with tap water.	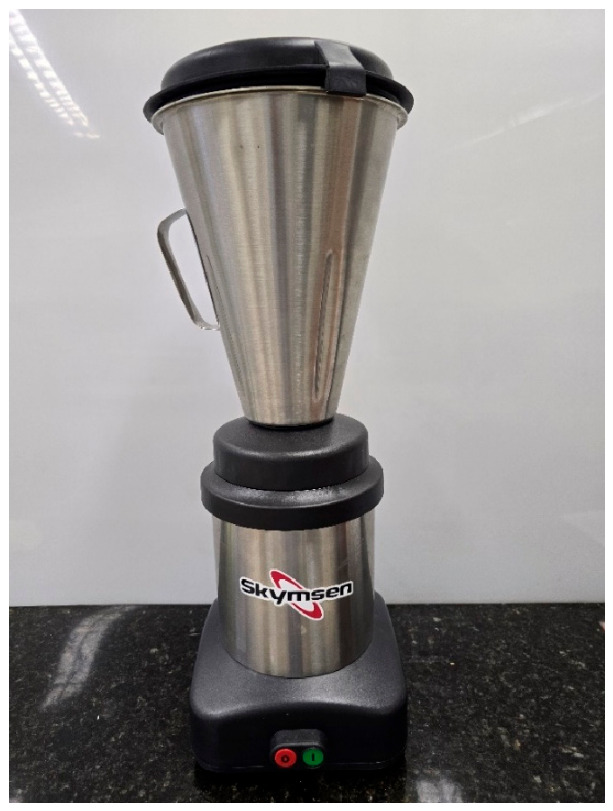
Watch glass	Used to hold or transfer small quantities of gel during preparation.	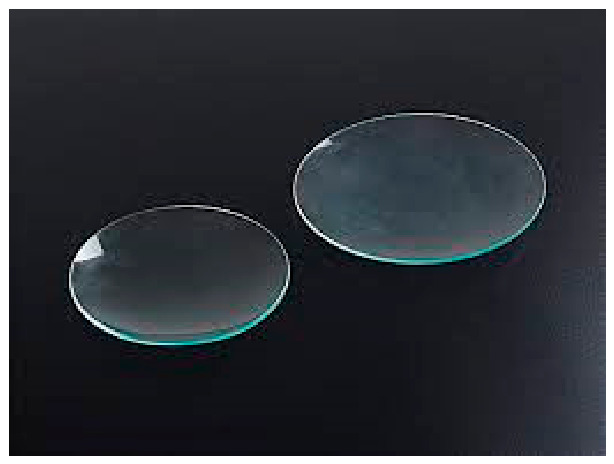
Beakers	Used to mix, hold, or measure the solution during the process.	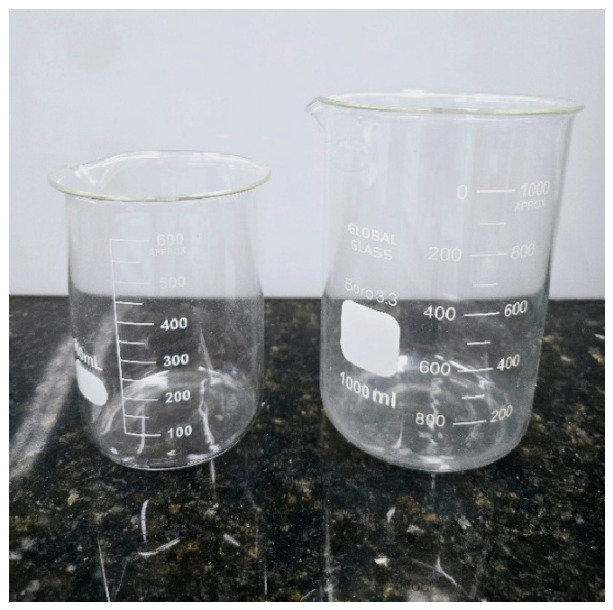
Balance	Used to weigh portions of gel for standardization.	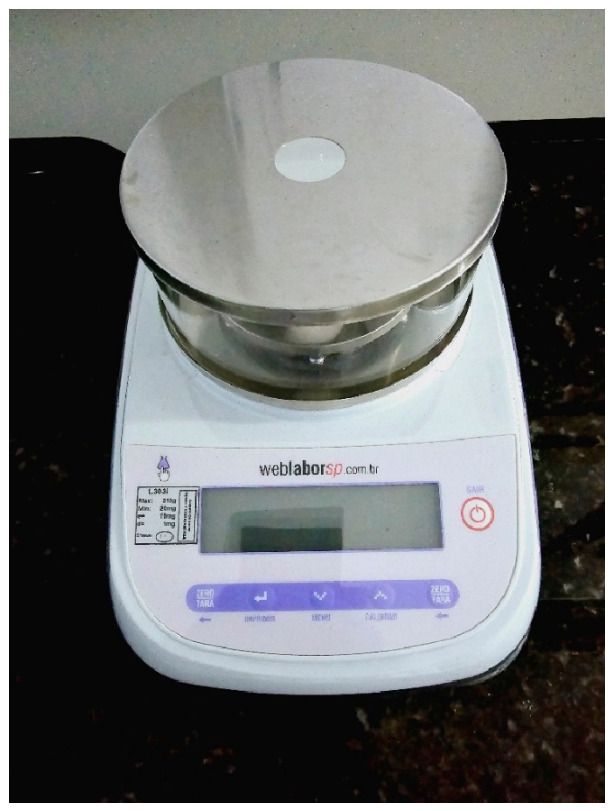
Spoon spatula	Used to scrape and transfer the extracted gel.	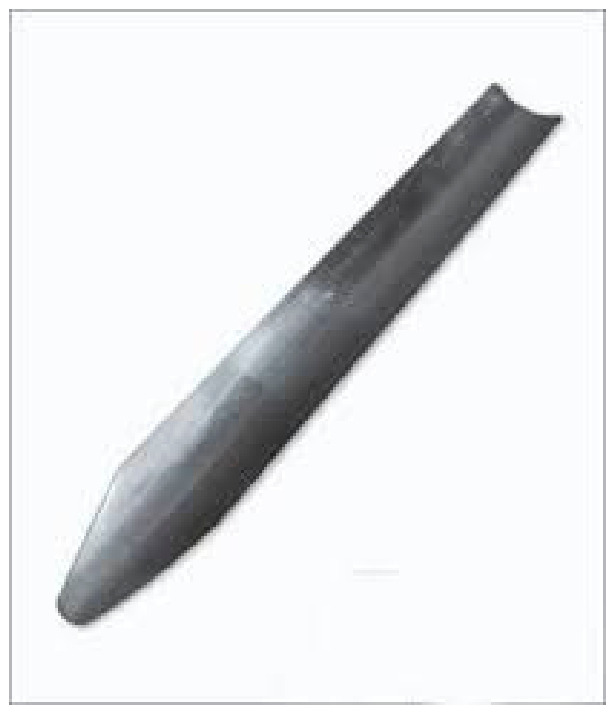
Knife	Used to cut and open the *Aloe vera* leaf.	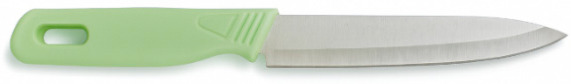

**Table 3:** Step-by-step protocol for preparing the *Aloe vera*-based natural coagulant.

Step	Description	Illustrative Image
Step 1	Selection of a healthy, mature *Aloe vera* leaf.	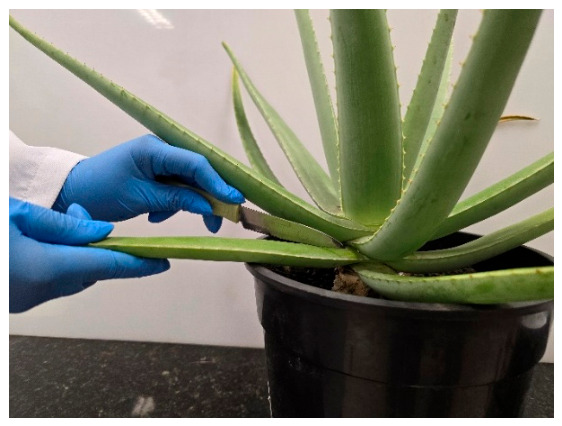
Step 2	Manual removal of a whole leaf from the base of the *Aloe vera* plant, preferably mature, using a clean and sharp knife.	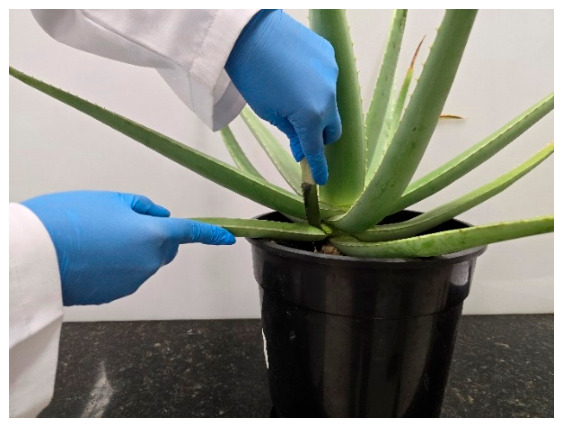
Step 3	After cutting, the leaf was placed at an angle over a beaker to allow the yellow liquid (aloin) to drain, as it is not used in the coagulation process.	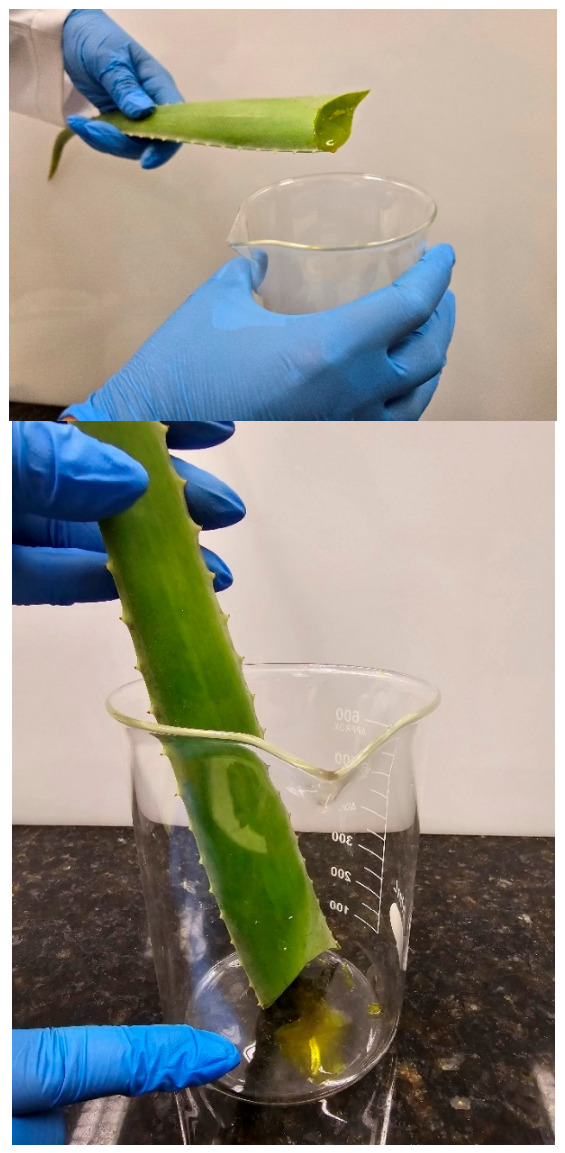
Step 4	The outer rind was carefully removed using a clean and sharp knife by making a longitudinal cut along the leaf to expose the inner gel.	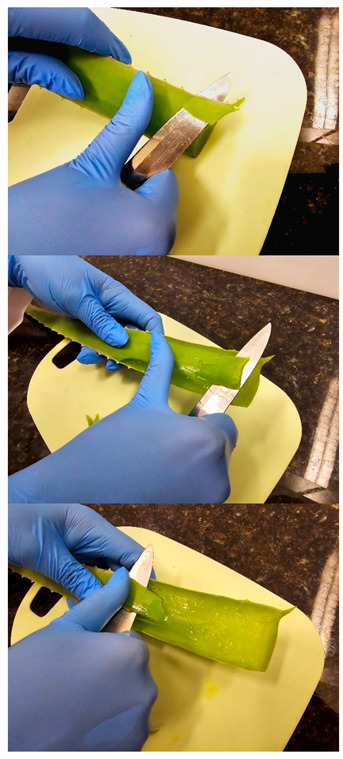
Step 5	The inner gel was extracted using a spoon spatula by gently sliding it along the inner surface of the opened leaf to completely remove the gel.	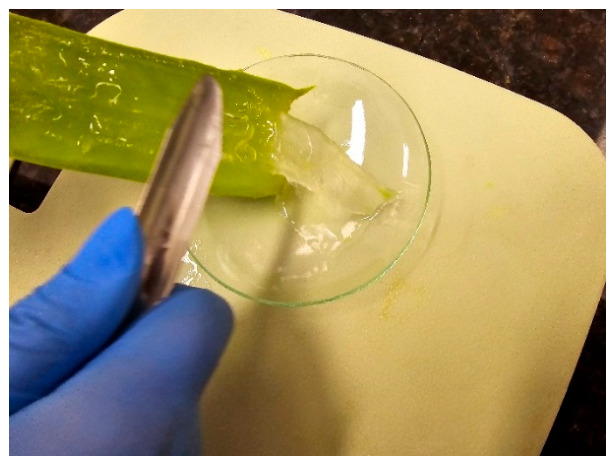
Step 6	The extracted gel was transferred to a pre-tared watch glass and weighed on an analytical balance (L303i, Weblabor, São Paulo, Brazil) to quantify the amount used in the coagulant preparation.	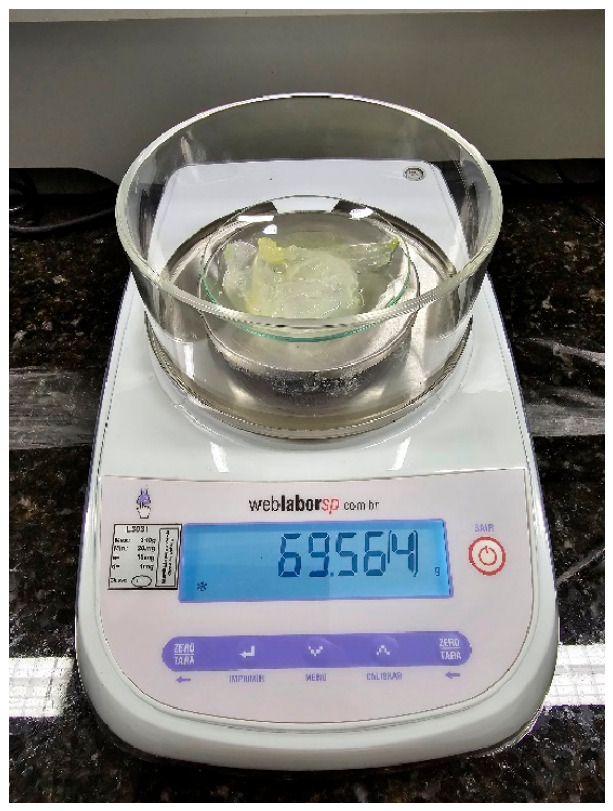
Step 7	The previously weighed gel was transferred to a household blender (Skymsen LC3, 500 W, 4500 rpm; Skymsen, Brusque, Brazil), 50 mL of tap water was added, and the mixture was homogenized for approximately 30 s. The gel-to-water ratio (2 g:50 mL), homogenization time, and blender operating conditions were maintained constant throughout all preparations to improve protocol reproducibility and facilitate replication of the proposed methodology.	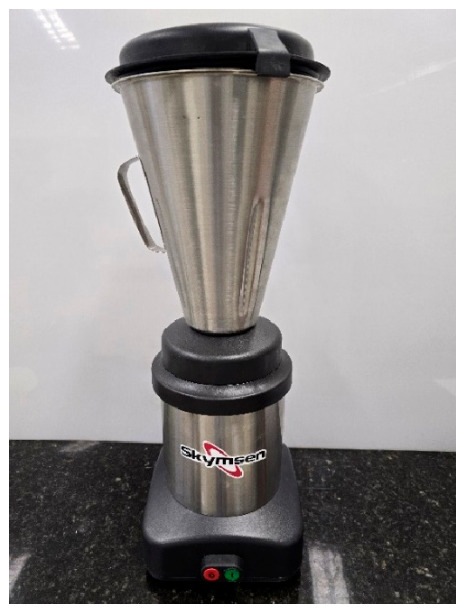
Step 8	After homogenization, the mixture was subjected to simple filtration using filter paper to remove residual solid particles and obtain the liquid coagulant.	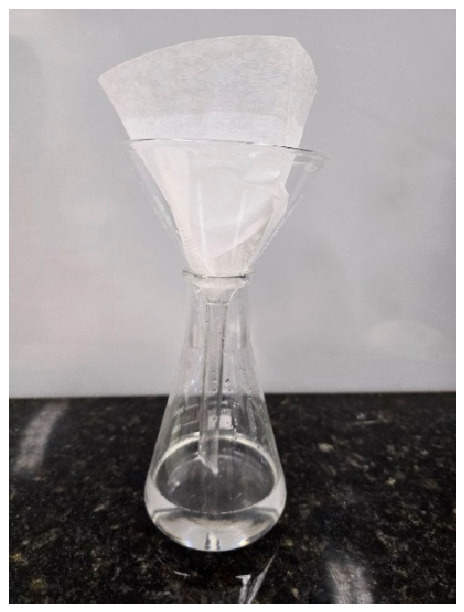
Step 9	The liquid coagulant obtained was transferred to a clean, properly labeled container for subsequent use in coagulation tests.	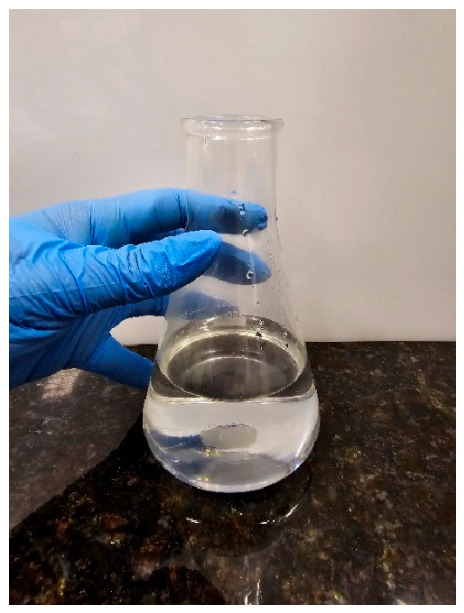

**Table 4:** Turbidity values for samples with an initial turbidity of approximately 300 NTU measured over settling times ranging from 0 to 60 min *.

Settling Time (min)	Turbidity (NTU)					
	Jar #1 (95 mL)	Jar #2 (100 mL)	Jar #3 (105 mL)	Jar #4 (110 mL)	Jar #5 (115 mL)	Jar #6 (120 mL)
0	314.00	312.67	313.00	311.00	313.67	311.00
10	32.00	32.37	25.44	25.39	19.89	16.67
20	14.96	13.18	8.01	7.33	3.82	2.02
30	11.21	8.88	7.34	6.17	4.07	0.00
40	8.90	9.37	6.11	4.08	2.78	0.00
50	6.61	5.00	1.80	1.44	0.00	0.00
60	6.61	5.00	1.80	1.44	0.00	0.00

* Values represent mean turbidity measurements obtained from triplicate experiments. Corresponding standard deviation values are provided in Appendix A.

**Table 5:** Turbidity values for samples with an initial turbidity of approximately 200 NTU measured over settling times ranging from 0 to 60 min *.

Settling Time (min)	Turbidity (NTU)					
	Jar #1 (80 mL)	Jar #2 (85 mL)	Jar #3 (90 mL)	Jar #4 (95 mL)	Jar #5 (100 mL)	Jar #6 (105 mL)
0	217.67	207.67	204.60	202.93	196.80	188.87
10	29.09	23.28	13.66	12.41	8.91	8.19
20	16.13	14.99	5.75	6.96	1.74	0.00
30	12.27	8.76	3.42	3.16	0.51	0.00
40	7.68	7.04	0.58	0.28	0.00	0.00
50	0.00	0.00	0.00	0.00	0.00	0.00
60	0.00	0.00	0.00	0.00	0.00	0.00

* Values represent mean turbidity measurements obtained from triplicate experiments. Corresponding standard deviation values are provided in Appendix A.

**Table 6:** Turbidity values for samples with an initial turbidity of approximately 100 NTU measured over settling times ranging from 0 to 60 min *.

Settling Time (min)	Turbidity (NTU)					
	Jar #1 (50 mL)	Jar #2 (55 mL)	Jar #3 (60 mL)	Jar #4 (65 mL)	Jar #5 (70 mL)	Jar #6 (75 mL)
0	101.77	98.37	96.97	99.40	91.37	89.93
10	25.46	20.49	18.50	16.28	17.18	14.21
20	10.81	10.17	7.87	5.28	6.28	4.09
30	3.44	2.48	1.26	0.00	0.24	0.24
40	3.82	1.61	0.64	0.00	1.38	0.00
50	1.66	0.00	0.00	0.00	0.00	0.00
60	0.00	0.00	0.00	0.00	0.00	0.00

* Values represent mean turbidity measurements obtained from triplicate experiments. Corresponding standard deviation values are provided in Appendix A.

## Data Availability

The data presented in this study are available on request from the corresponding author.

## Appendix A. Standard Deviation Values Obtained from Triplicate Measurements

To provide additional information regarding experimental variability and result reproducibility, the standard deviation values calculated from triplicate measurements are presented in this appendix. Standard deviations were determined for all evaluated turbidity levels (100, 200, and 300 NTU), coagulant dosages, and settling times. These data complement the mean turbidity values presented in Table A1, Table A2 and Table A3 of the main manuscript and provide an estimate of the consistency of the proposed *Aloe vera*-based coagulant preparation protocol.

**Table A1:** Standard deviation values (NTU) obtained from triplicate measurements for samples with an initial turbidity of approximately 100 NTU *.

Settling Time (min)	Jar #1 (50 mL)	Jar #2 (55 mL)	Jar #3 (60 mL)	Jar #4 (65 mL)	Jar #5 (70 mL)	Jar #6 (75 mL)
0	1.06	10.01	13.91	10.36	7.15	4.40
10	6.71	9.16	8.64	9.82	7.04	10.04
20	1.81	6.82	2.85	4.57	5.80	3.05
30	2.53	0.47	0.88	0.00	0.42	0.42
40	1.03	1.46	1.11	0.00	1.83	0.00
50	0.33	0.00	0.00	0.00	0.00	0.00
60	0.00	0.00	0.00	0.00	0.00	0.00

**Table A2:** Standard deviation values (NTU) obtained from triplicate measurements for samples with an initial turbidity of approximately 200 NTU *.

Settling Time (min)	Jar #1 (80 mL)	Jar #2 (85 mL)	Jar #3 (90 mL)	Jar #4 (95 mL)	Jar #5 (100 mL)	Jar #6 (105 mL)
0	7.23	5.13	12.98	16.52	1.11	9.22
10	12.86	9.27	10.43	10.87	11.52	10.76
20	2.47	3.29	1.60	2.18	2.03	0.00
30	3.29	1.65	2.66	3.23	0.44	0.00
40	0.45	1.00	0.18	0.49	0.00	0.00
50	0.00	0.00	0.00	0.00	0.00	0.00
60	0.00	0.00	0.00	0.00	0.00	0.00

**Table A3:** Standard deviation values (NTU) obtained from triplicate measurements for samples with an initial turbidity of approximately 300 NTU *.

Settling Time (min)	Jar #1 (95 mL)	Jar #2 (100 mL)	Jar #3 (105 mL)	Jar #4 (110 mL)	Jar #5 (115 mL)	Jar #6 (120 mL)
0	13.23	10.69	8.72	8.89	11.02	3.61
10	16.67	14.53	11.10	12.20	13.07	13.93
20	4.39	4.65	2.97	1.08	3.36	1.87
30	4.80	1.45	2.96	4.01	2.83	0.00
40	3.15	1.75	1.40	1.03	0.77	0.00
50	1.66	3.13	2.30	1.93	0.00	0.00
60	0.68	0.56	0.00	0.80	0.00	0.00

* Standard deviation values were calculated from triplicate measurements performed for each experimental condition. Higher variability was generally observed during the initial settling stages, while lower standard deviation values were obtained at longer settling times, indicating increasing stability of the clarification process and satisfactory consistency of the proposed coagulant preparation protocol.
